# Does the early aldosterone‐induced SGK1 play a role in early Kaliuresis?

**DOI:** 10.14814/phy2.15188

**Published:** 2022-02-28

**Authors:** Lama Al‐Qusairi, Denis Basquin, Matteo Stifanelli, Paul A. Welling, Olivier Staub

**Affiliations:** ^1^ Division of Nephrology Johns Hopkins University School of Medicine Baltimore USA; ^2^ Department of Physiology University of Maryland Baltimore USA; ^3^ Department of Biomedical Sciences University of Lausanne Lausanne Switzerland

## Abstract

Urinary K^+^ potassium excretion rapidly increases after a potassium‐rich meal. The early aldosterone‐induced *sgk1* gene (encoding serum and glucocorticoid‐induced kinase 1), activates potassium clearance, but the role of this kinase in the early activation of K^+^ secretion has not been clearly defined. Here, we challenged inducible renal‐tubule‐specific *Sgk1^Pax8^* ^/^ *^LC1^* knockout mice with an acute high‐potassium load (HK:5%K^+^) and compared the physiological and molecular responses to control mice. We observe that urinary excretion after a K^+^ load over the first 3 h is not dependent on SGK1 but is coincident with the rapid dephosphorylation of the Na^+^,Cl^−^‐cotransporter (NCC) to increase distal salt delivery. Molecular analyses indicate that whereas SGK1‐mediated phosphorylation of the ubiquitin‐protein ligase NEDD4‐2 begins to increase by 3h, SGK1‐dependent proteolytic activation of ENaC only becomes detectable after 6 h of HK intake. Consistent with SGK1‐dependent ENaC activation via inhibition of NEDD4‐2‐mediated ubiquitylation, *Sgk1^Pax8^* ^/^ *^LC1^* mice are unable to efficiently inhibit NEDD4‐2 or increase ENaC cleavage after 6 h of HK. Nevertheless, no defect in acute K^+^ balance was detected in the mutant mice after 6 h of HK. Moreover, we found that *Sgk1^Pax8^* ^/^ *^LC1^* mice reduce NCC phosphorylation and NCC‐mediated salt absorption to a greater extent than control mice after a K^+^ load, promoting increased amiloride‐sensitive Na^+^‐reabsorption via ENaC to maintain adequate kaliuresis. Together, these data indicate that: (a) during the early 3 h of HK intake, K^+^ excretion is SGK1‐independent even under an extreme K^+^ challenge, (b) shortly after, SGK1 inhibits NEDD4‐2 and activates ENaC to stimulate K^+^‐secretion, (c) SGK1‐dependent phosphorylation of NCC occurs, acting more likely as a brake pedal to prevent excessive K^+^ loss.

## Graphical abstract

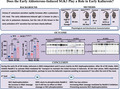

## 1 INTRODUCTION

The role of serum and glucocorticoid‐induced kinase (SGK1) and its target, the ubiquitin‐protein ligase NEDD4‐2, in the long‐term control (days to weeks) of potassium homeostasis is well accepted (Al‐Qusairi et al., 2016, 2017; Debonneville et al., 2001; Faresse et al., 2012; Huang et al., 2004; Kamynina et al., 2001, 2001). SGK1 and NEDD4‐2 orchestrate the function of channels and transporters in the distal nephron that directly or indirectly influence K^+^ homeostasis, including ENaC (Al‐Qusairi et al., 2016, 2017; Huang et al., 2004), NCC (Arroyo et al., 2011; Ronzaud et al., 2013), and the Kir4.1/Kir5.1 channel (Wang et al., 2018; Wu et al., 2020).

SGK1 increases K^+^ secretion, in part, by phosphorylating NEDD4‐2 and activating ENaC‐mediated Na^+^ transport (Alvarez de la Rosa et al., 1999; Lang et al., 2000). NEDD4‐2 is a ubiquitin‐protein ligase that negatively regulates ENaC. Upon binding to the ENaC PY‐motif, NEDD4‐2 ubiquitylates the channel and induces its internalization (Abriel et al., 1999; Staub et al., 1997). In high aldosterone states of dietary Na^+^ deficiency or K^+^ excess, SGK1 phosphorylates NEDD4‐2 at three serine residues, Ser 222, Ser 246, and Ser 328 (Bhalla et al., 2005; Debonneville et al., 2001; Snyder et al., 2002), thereby preventing the ubiquitin‐protein ligase from interacting with ENaC. In inducible, kidney‐tubule‐specific *Sgk1* knockout mice, NEDD4‐2 is not efficiently phosphorylated in high aldosterone states, compromising aldosterone‐dependent increase in ENaC apical expression and/or proteolytic processing. A similar response has been described in mice with germ‐line mutation (SGK1^−/−^) (Al‐Qusairi et al., 2016; Faresse et al., 2012; Huang et al., 2004; Yang et al., 2017). The opposite phenotype is observed in *Nedd4*‐*2* KO mice, which increased ENaC expression and activity (Boase et al., 2011; Shi et al., 2008), even when aldosterone is suppressed in dietary potassium deficiency (Al‐Qusairi et al., 2017).

The thiazide‐sensitive Na^+^, Cl^−^‐cotransporter, NCC, in the distal convoluted tubule is also a target of SGK1/NEDD4‐2 regulation (Al‐Qusairi et al., 2016, 2017; Arroyo et al., 2011; Faresse et al., 2012; Ronzaud et al., 2013; Vallon et al., 2009; Wu et al., 2020), likely controlling distal sodium delivery to indirectly regulate K^+^ secretion in the ASDN. According to present understanding, SGK1 blocks NEDD4‐2 from negatively regulating NCC. Consistent with this idea, NCC abundance and phosphorylation are decreased in *Sgk1* knockout and increased in NEDD4‐2 knockout mice (Al‐Qusairi et al., 2016, 2017; Faresse et al., 2012; Ronzaud et al., 2013). Moreover, SGK1/NEDD4‐2 pathway has been shown to negatively regulate a PY‐motif containing L‐WNK1 isoform, involved in NCC phosphorylation cascade (Roy et al., 2015). At present, it is unclear if these effects are direct or indirect because SGK1/NEDD4‐2 can affect plasma potassium levels, which in turn affect abundance, phosphorylation, and activity of NCC (Al‐Qusairi et al., 2016; Huang et al., 2004; Vallon et al., 2009).

*Sgk1* is one of the earliest aldosterone (Aldo)‐induced genes, detected as early as 2–4 h after Aldo treatment in kidneys of adrenalectomized (ADX) rat (Chen et al., 1999; Loffing et al., 2001). Despite extensive studies describing its role in long‐term K^+^ homeostasis, our knowledge about the early role of SGK1 in urinary K^+^ excretion is lacking. Early studies have reported that acute K^+^ load induced rapid kaliuresis in animals and human (Buren et al., 1992; Rabinowitz et al., 1985). Elegant studies from Loffing's group revealed that the rapid kaliuresis is mediated by an aldosterone‐independent pathway, mostly driven by NCC dephosphorylation, followed by a late aldosterone‐dependent phase characterized by ENaC synthesis and proteolytic activation (Sorensen et al., 2013). Little is known about the specific role of SGK1 in this early kaliuresis. Interestingly, mice harboring a germ‐line SGK1 deletion are unable to efficiently increase K^+^ excretion assessed 1h after acute intravenous K^+^ load (Huang et al., 2004). However, it is not clear if this defect is due to renal or extrarenal SGK1 deletion (Lang & Vallon, 2012), or it is due to the chronic lifetime loss of renal SGK1 that alters the ability of the kidney to respond to acute K^+^ load.

The current study explores the role of SGK1 in short‐term K^+^ regulation. Physiological and biochemical responses to an acute K^+^ load were studied in inducible kidney‐tubule‐specific *Sgk1* knockout and control littermate mice. We found that the early kaliuresis occurs independently of SGK1 during the first 3 h of high‐K^+^ intake, and the role of SGK1 in the early kaliuresis starts only after 3 h of K^+^ loading by phosphorylating and inhibiting NEDD4‐2 leading to subsequent increase in ENaC to stimulate potassium secretion. Our data revealed a SGK1‐dependent phosphorylation of NCC which might act as a brake pedal to prevent excessive K^+^ loss.

## 2 MATERIALS AND METHODS

### 2.1 Animals

Inducible, renal‐tubule‐specific *SGK1^flox^* ^/^ *^flox^*/*Pax8*‐*rTA*/*LC1* knockout *(SGK1^Pax8^* ^/^ *^LC1^)* or control littermate *Sgk1^Pax8^* or *Sgk1^LC1^* mice were housed in a temperature‐controlled facility (19–22°C) with a 12:12‐h light‐dark cycle. To induce gene deletion, 21 to 24 day‐old mice were treated with doxycycline (2 mg/ml in 2% sucrose in drinking water) for 12 days as previously described (Al‐Qusairi et al., 2016; Faresse et al., 2012). The status of gene deletion has been verified in all the mice included in this study using PCR on genomic DNA extracted from total kidney as previously described (Al‐Qusairi et al., 2016; Faresse et al., 2012). After 2 days of wash‐out, age‐matched males (7‐8 weeks) were subjected to experimentation. Experimental protocols were designed with respect to the Swiss Animal Welfare Act and approved by the veterinary administration of the Canton de Vaud (Switzerland); authorization number: 2590.

### 2.2 Acute K^+^ loading by gastric gavage

Gastric gavage was performed as previously described (Sorensen et al., 2013). Briefly, animals were subjected to gastric gavage with solutions containing 2% sucrose (control solution) or 2% sucrose +2% K+ (512 mM) with HCO3‐ as anion to mimic the K_3_‐citrate used as a K^+^ supplement in the diet. We used KHCO3 since the physiologic and molecular responses to KHCO3 has been extensively characterized by Sorensen et al. (2013). Urine samples were collected before gavage (basal) and after 30 min of gavage (30 min HK). 30 min after gavage, mice were euthanized to collect blood and organs for further analysis.

### 2.3 Dietary manipulation

Control and Sgk1^Pax8/LC1^ doxycycline treated‐mice were fed a standard diet (1% K^+^, Ssniff) or HKD (5% K^+^ with K_3_‐citrate used as a K^+^ supplement, Ssniff) for the periods indicated. To decrease variability in food intake over a short period of feeding (3 and 6 hours), a food‐restriction period of 10–12 h was applied before the feeding experiment. Animals were acclimated for 3 days to food restriction periods. No difference in food intake was observed between mutant and control mice for the experimented periods (Figure S1a,b). To conserve circadian rhythmicity of SGK1 expression and electrolyte handling (Gumz, 2016), the food restriction period was applied during the inactive phase while the feeding period was applied at the beginning of the active phase. Mice were kept in circadian boxes designed for this purpose. Plasma K^+^, urinary K^+^, and immunoblot data are obtained from the same animals for each time point, a different cohort was used at each time point.

### 2.4 Diuretic treatment and metabolic cages

**FIGURE 1:**
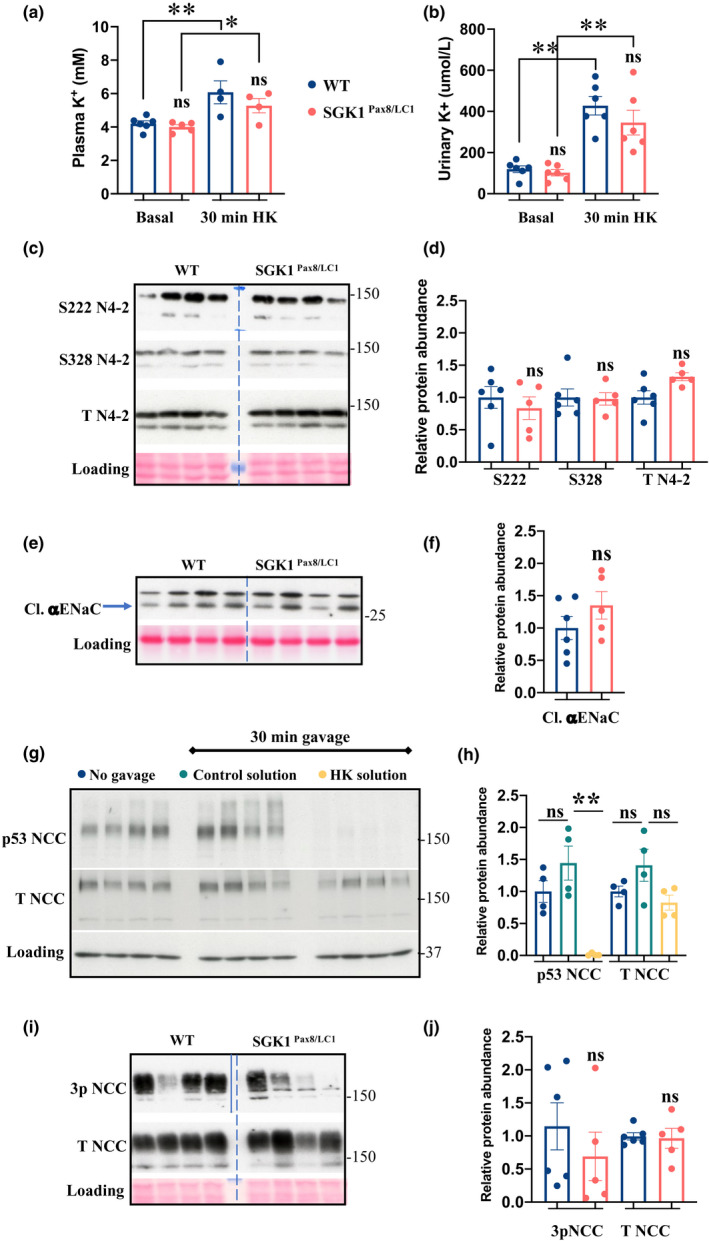
*sgk1^Pax8^* ^/^ *^LC1^* KO mice conserve normal ability to handle acute K^+^ load. (a) Plasma K^+^ under control diet and 30 min post gavage, *N* = 4/group. (b) Urinary K^+^ under basal and 30 min post gavage with HK solution (2% sucrose +2% K^+^) showing a normal increase in K^+^ clearance in the mutant mice, *N =* 6/group. (c) Western blot (WB) analysis of total kidney lysate from control and mutant mice 30 min after gavage with HK showing total (T N4‐2) and serine phosphorylated levels of N4‐2. (d) Quantification of (c). (e) WB analysis showing normal processing of αENaC in the mutant mice 30 min after gavage. (f) Quantification of (e). (g) WB analysis showing sharp decrease in NCC phosphorylation in WT mice 30 min post gavage with HK solution compared to control solution (sucrose) or to the no‐gavage group that was included in the experiment to exclude a stress‐induced effect. (h) Quantification of (g). (i) WB analysis showing similar NCC dephosphorylation in the mutant and control littermates 30 min post gavage. (j) Quantification of (i). For WB analysis: *N =* 4–6 animals/group. Data in the dots plot are all obtained from the same WB experiments, representative images of these WBs are presented above. Two‐way ANOVA was used to assess the significance in a, b, and h, two‐tailed unpaired *t*‐test was used in the other analyses. **: p value < 0.001, ns: non‐significant

**FIGURE 2:**
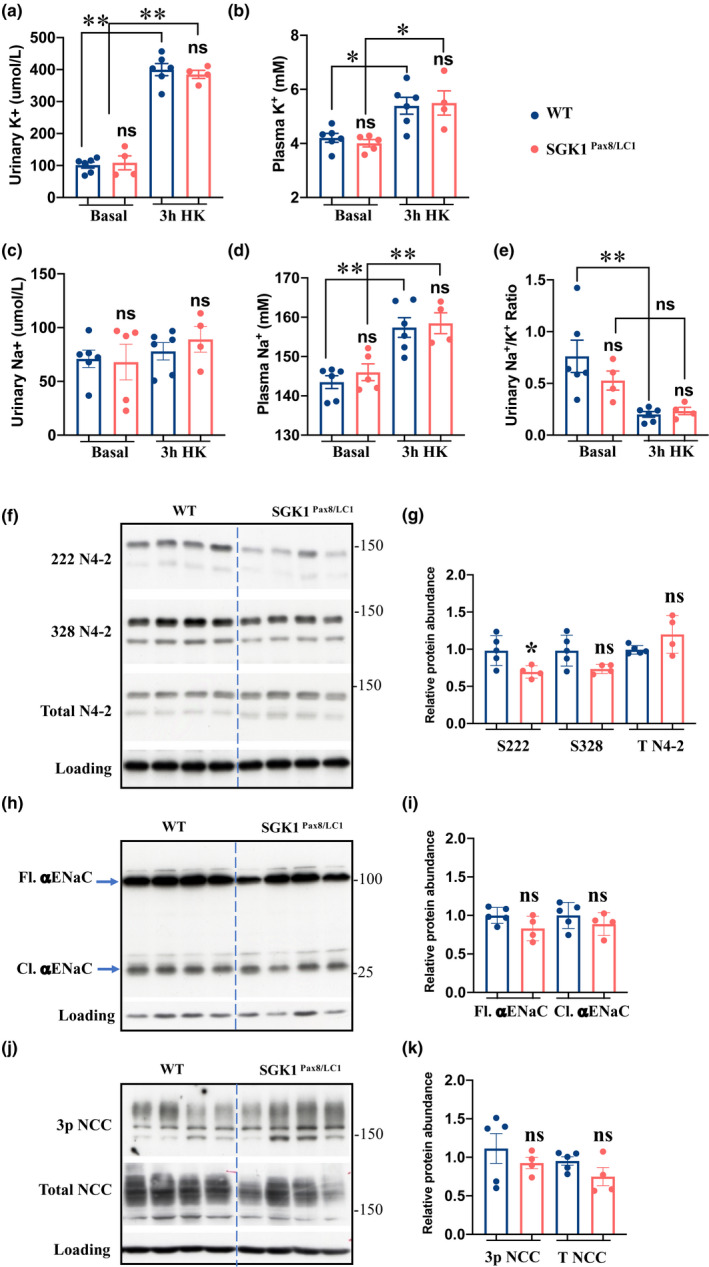
Renal‐SGK1 is involved in N4‐2 phosphorylation as early as 3 h after HKD intake. (a,b) Urinary and plasma K^+^ at basal levels and 3 h after the start of HKD intake, basal plasma K^+^ levels are replotted from 1a to facilitate comparison. (c,d) Urinary and plasma Na^+^ at basal levels and 3 h after the start of HKD intake. (e) Urinary Na^+^/K^+^ ratio at basal levels and 3 h after the start of HKD. (f) WB analysis of total kidney lysates from mutant and control littermates at 3 h after the start of HK intake showing total and serine phosphorylated N4‐2. (g) Quantification of (f). (h) WB analysis of αENaC showing both the full length (Fl.) and cleaved (Cl.) forms of the α subunit at 3 h after the start of HK intake. (i) Quantification of (h). (j) WB analysis of total NCC and 3p NCC at 3 h after the start of HK intake. (k) Quantification of WB in (j). *N* = 4–5/group. Data in the dots plot are all obtained from the same WB experiments, representative images of these WBs are presented above. Two‐way ANOVA was used to assess the significance in (a‐d), two‐tailed unpaired *t*‐test was used in the other analyses. **:*p* value < 0.001, *:*p* value < 0.05, ns: non‐significant

**FIGURE 3:**
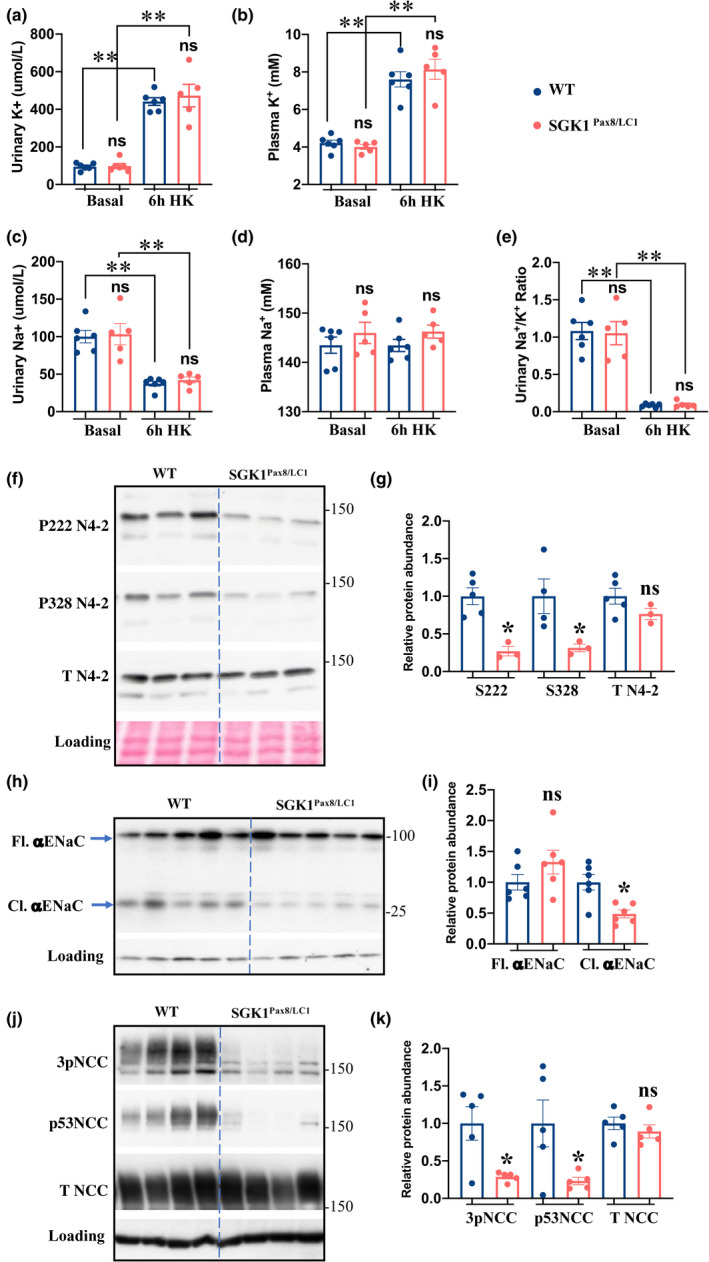
ENaC and NCC regulation is altered in SGK1^Pax8/LC1^ as early as 6 h after the start of HK intake. (a,b) Urinary and plasma K^+^ at basal levels and 6 h after the start of HKD intake, basal plasma K^+^ levels are replotted from 1a to facilitate comparison. (c,d) Urinary and plasma Na^+^ at basal levels and 6 h after the start of HKD intake. (e) Urinary Na^+^/K^+^ ratio at basal levels and 6 h after the start of HKD. (f) WB analysis of total kidney lysates from mutant and control littermates after 6 h of HK intake showing total and serine phosphorylated N4‐2, *N* = 5WT and 3 KO. (g) Quantification of (f). (h) WB analysis of αENaC showing both the full length (Fl.) and cleaved (Cl.) forms of the α subunit, *N* = 6 animals/group. (i) Quantification of (h). (j) WB analysis of total and phosphorylated NCC, *N* = 5/group. (k) Quantification of WB in (j). Data in the dots plot are all obtained from the same WB experiments, representative images of these WBs are presented above. Two‐way ANOVA was used to assess the significance in (a‐d) two‐tailed unpaired *t*‐test was used in the other analyses. **:*p* value < 0.001, *:*p* value < 0.05, ns: non‐significant

After 2 days of adaptation in metabolic cages (catalog no. 3600M021, Indulab), mice were injected IP with amiloride (5 mg/kg BW), thiazide (20 mg/kg BW), or vehicle (DMSO). After 6 h diet change, diuretics were injected at 6h, and urine was collected between 6 and 12 h after diet change. The timing of diuretics treatment 6–12 h after diet change was chosen because the data from Figures 1, 2, 3 have shown no deregulation in NCC and ENaC are detected up to 3 h. Electrolyte levels were measured with a flame photometer (Cole‐Palmer Instrument).

### 2.5 Plasma and organs collection

Mice were anesthetized by a ketamine/xylazine intraperitoneal (IP) injection. Blood was collected from the retro‐orbital plexus in SARSTEDT heparin‐containing microtubes, and plasma was separated according to the manufacturer's instructions. After kidney collection, animals were euthanized by cervical dislocation. Plasma Na^+^ and K^+^ were measured using a flame photometer (Cole‐Palmer Instrument).

Basal plasma K^+^ was measured in one group of mice that was not subjected to food restriction and represented in Figures 1a, 2b, 3b. It is worth mentioning that basal plasma K^+^ might exhibit differences between non‐food restriction and food restriction followed be refeeding (3 h or 6 h with 1% K^+^) However, it is unlikely that these minor differences impact the final outcome of this study.

### 2.6 Protein lysate preparation and immunoblot analysis

Frozen tissues were homogenized using buffer containing 50 mM Tris·HCl (pH7.5), 1 mM EDTA, 1 mM EGTA, 0.27 M sucrose, 50 mM NaF, and 5 mM Na‐pyrophosphate in addition to protease inhibitors purchased from Roche (Complete catalog no. 11836145001). Protein homogenates were then centrifuged at 10,000 *g* for 10 min at 4°C. The supernatant was collected, and protein concentration was measured using the Bradford method (catalog no. UPF86420, Uptima). Proteins were separated by SDS–PAGE and transferred to nitrocellulose membranes. After 1h blocking with non‐fat milk, membranes were incubated overnight with the primary antibodies at 4^o^C, washed for 1 h with TBS 2% tween, incubated 2 h with the secondary antibody, and washed for another 1h with TBS 2% tween. A list of the antibodies used in this study is presented Table 1.

**TABLE 1:** List of the primary antibodies used in this study

Antibody	Host	WB dilution	Reference
3pNCC S(45–55–60)	Sheep	1/700	Kindly provided by Dario Alessi
pT53 NCC	Rabbit	1/500	Sorensen MV., Kidney international 2013
Total NCC	Rabbit	1/500	Sorensen MV., Kidney international 2013
αENaC	Rabbit	1/500	Sorensen MV., Kidney international 2013
pS222 NEDD4‐2	Sheep	1/600	Faresse N, AJP‐renal Physiol 2012
pS328 NEDD4‐2	Rabbit	1/500	Flores S., JASN 2005
Total NEDD4‐2	Rabbit	1/1000	Kamynina E., FASEB J 2001
GAPDH	Mouse	1/5000	Millipore (MAB374)

### 2.7 Statistical analysis

Graphs and statistical analyses were performed using GraphPad PRISM version 8. The significance of the data was assessed using unpaired *t*‐test or two‐way ANOVA as indicated for each experiment in the legends. Correction for multiple‐comparison was performed using Holm–Sidak method. Values were considered significant at *p* < 0.05. Data are represented as means ± SEM.

## 3 RESULTS

### 3.1 *Sgk1^Pax8^* ^/^ *^LC1^* mice efficiently excrete K^+^ after an acute K^+^ load

Here, we studied the response of kidney‐tubule‐specific *Sgk1* knockout mice to acute K^+^ loading. Gastric gavage was used since it has been shown to mimic high K^+^ feeding (Sorensen et al., 2013), and the molecular mechanism of kaliuresis has been described (Sorensen et al., 2013; Terker et al., 2015). *Sgk1^Pax8^* ^/^ *^LC1^* increased plasma levels (Figure 1a) and urinary K^+^ concentration to the same extent as control mice 30 min post K^+^ loading (Figure 1b). Neither NEDD4‐2 phosphorylation (S222 and S328) (Figure 1c,d), nor αENaC cleavage, a marker of ENaC activation (Figure 1e,f), were affected by SGK1 deletion at 30 min after gavage. Furthermore, based on Sorensen's studies, the early aldosterone‐independent phase is mediated by NCC dephosphorylation which is detected as early as 15 min after HK gavage (Sorensen et al., 2013). We first confirmed this finding in WT mice showing 30 min HK gavage induces a sharp decrease in NCC phosphorylation compared to control gavage (sucrose only) or to no‐gavage groups (Figure 1g,h). We then evaluated NCC phosphorylation in *Sgk1^Pax8^* ^/^ *^LC1^* and control littermate after 30 min gavage and found the same level of NCC phosphorylation in both groups, suggesting that NCC was dephosphorylated by high‐K^+^ intake to similar extent in both genotypes (Figure 1i,j). Together these data indicate renal SGK1 is not necessary for the rapid kaliuresis induced by acute K^+^ load.

### 3.2 II‐A functional effect of SGK1 is observed as early as 3h after high K^+^ load

The aldosterone‐dependent phase of Na^+^ reabsorption includes an early phase (2–3 h after a lag period) characterized by the stimulation of the preexisting Na^+^ reabsorption machinery in the distal nephron (Bachmann et al., 1999; Loffing et al., 2001; Meneton et al., 2004). To investigate the role of renal SGK1 in urinary potassium excretion during the early aldosterone response, we fed *Sgk1^Pax8^* ^/^ *^LC1^* and control mice HK diet (5%K) for 3 h; similar food intake was observed in both genotypes (Figure S1). We found that both groups increased urinary K^+^ concentration by about 4 times after HK feeding compared to basal urinary K^+^ (Figure 2a). Similarly, both genotypes were slightly hyperkalemic after 3 h HK intake compared to control diet with no difference between *Sgk1^Pax8^* ^/^ *^LC1^* and control mice (Figure 2b). We also found no difference in urinary Na^+^ concentration between basal and 3 h HK intake, similar observation in both genotype (Figure 2c). Moreover, both mutant and control mice exhibit increased plasma Na^+^ after 3 h of HK intake (Figure 2d). Na^+^/K^+^ ratio was lower at 3h HK intake compared to basal levels with no difference between genotypes (Figure 2e). NEDD4‐2 S222 but not S328 phosphorylation was decreased in the *Sgk1^Pax8^* ^/^ *^LC1^* vs. control animals (Figure 2f,g). αENaC was similarly expressed and cleaved in both genotype (Figure 2h,i). No difference in NCC phosphorylation or total protein levels were observed between the two groups, suggesting that high‐K^+^‐mediated NCC dephosphorylation was similar in both genotypes (Figure 2j,k). These data indicate that after 3 h ingestion of a HKD, SGK1 begins to exert a regulatory effect on NEDD4‐2 phosphorylation before changes in ENaC cleavage can be detected.

### 3.3 The aldosterone‐dependent phase requires SGK1 to regulate ENaC and NCC

The analysis of *Sgk1^Pax8^* ^/^ *^LC1^* and control mice at later time‐point reveals that, for similar food intake (Figure S1b), both genotypes increase their urinary K^+^ concentration by about 4 to 4.5 times after 6 h of the start of HK intake with no significant difference between genotypes (Figure 3a). Compared to basal levels (4.2 in controls vs. 4.0 in mutants), plasma K^+^ rise to (7.6 mM ± 1 in controls vs. 8.1 mM ± 1.2 in mutants), with no significant difference between the genotypes (Figure 3b). The observed kaliuresis was accompanied by a decrease in urinary Na^+^, which occurs to similar extent in both genotype (Figure 3c). Interestingly, the increase in plasma Na^+^, observed at 3h, returned to basal levels in both genotypes (Figure 3d). Na^+^/K^+^ ratio was lower at 6h HK intake compared to basal levels with no difference between genotypes (Figure 3e).

**FIGURE 4:**
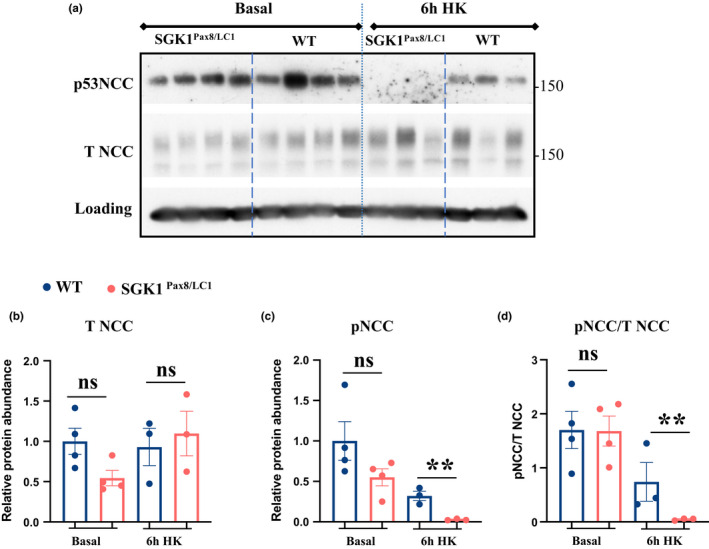
NCC dephosphorylation is more pronounced in the SGK1^Pax8/LC1^ compared to WT mice despite similar plasma K^+^. (a) WB analysis of phosphorylated and total NCC in WT and mutated mice under basal and 6h after the start of HK diet. (b,c) Quantification of protein abundance in (a) relative to loading control. (d) Ratio of phosphorylated to total NCC from (A). *N* = 3–4 mice/group. Two‐Way ANOVA was used to assess significance, **:*p* value < 0.001, *:*p* value < 0.05, ns: non‐significant

In the absence of SGK1, NEDD4‐2 serine residues S222 and S328 are not efficiently phosphorylated after 6 h ingestion of the HK diet (Figure 3f,g). Failure to suppress NEDD4‐2 binding to ENaC is correlated with a defect in the proteolytic processing of the αENaC subunit, as evidenced by reduced abundance of cleaved αENaC in the *Sgk1^Pax8^* ^/^ *^LC1^* mice (Figure 3h,i). Interestingly, despite similar plasma K^+^ concentration, NCC becomes more dephosphorylated in *Sgk1* KO compared to control mice (Figure 3j,k). To ensure that this defect was not present at a basal K^+^ intake, we assessed NCC phosphorylation in mutant vs. control mice under basal and after 6 h of HK intake. As shown in (Figure 4a–d), no difference in NCC phosphorylation nor total expression was observed between the two genotypes under basal K^+^ intake. As expected, NCC becomes dephosphorylated upon HK intake in both genotypes but, the dephosphorylation was more enhanced in *Sgk1^Pax8^* ^/^ *^LC1^* compared to the control mice (Figure 4a‐d), despite similar increases in plasma K^+^ after 6 h. This indicates that, during K^+^ secretion, SGK1 plays a role in preventing excessive NCC dephosphorylation.

Together, our data indicate the aldosterone‐dependent phase of K^+^ secretion requires SGK1 to upregulate ENaC processing. At the same time, SGK1‐dependent processes suppress NCC dephosphorylation, perhaps to tune the kaliuretic response avoiding an excessive K^+^ loss after the end of the meal.

### 3.4 SGK1 deletion decreases HCTZ‐sensitive and increases amiloride‐sensitive Na^+^ excretion

**FIGURE 5:**
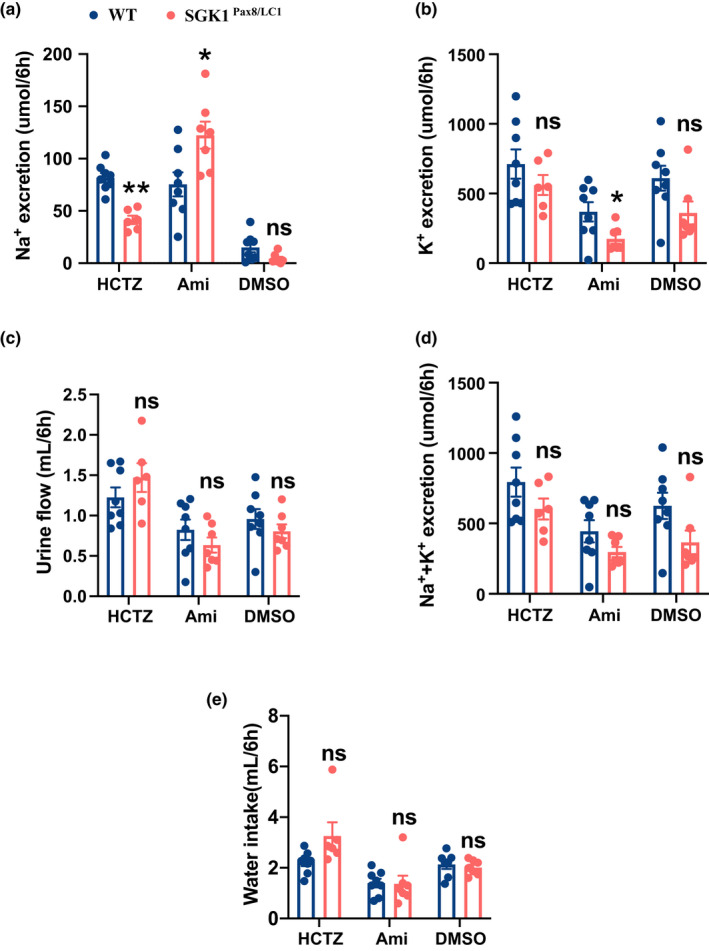
Renal SGK1 deletion results in increased amiloride‐sensitive and decreased thiazide‐sensitive Na^+^‐secretion. (a) Urinary Na^+^ measurement showing decreased HCTZ‐sensitive and increased amiloride‐sensitive Na^+^ secretion in the SGK1^Pax8/LC1^ vs. control mice. (b) Urinary K^+^ measurement showing a tendency for decreased K^+^ secretion in the mutant vs. control mice which becomes significant only upon amiloride treatment. (d) Urinary (Na^+^ +K^+^) excretion in the mutant and control mice. (c,e) Urinary flow and water intake were similar between mutant and control mice. Multiple T‐Tests were used to assess significance, multiple comparison was corrected for using Holm–Sidak method. **:*p* value < 0.001, *:*p* value < 0.05, ns: non‐significant

To test the functional consequences of SGK1‐dependent ENaC cleavage and NCC phosphorylation after 6 h consumption of a K^+^ load, we measured the urinary responses of *Sgk1^Pax8^* ^/^ *^LC1^* and control mice under HK diet to a single dose of Hydrochloride Thiazide (HCTZ: 20 mg/kg BW), or amiloride (Ami: 5 mg/kg BW). After 6 h of vehicle (DMSO) injection, urinary Na^+^ and K^+^ excretion was similar in both genotypes (Figure 5a,b). Interestingly, HCTZ‐induced natriuresis was reduced in *Sgk1^Pax8^* ^/^ *^LC1^* vs. control mice, while no difference in K^+^ excretion was found between both genotypes upon HCTZ treatment (Figure 5a,b). Amiloride‐induced natriuresis was greater in the *Sgk1^Pax8^* ^/^ *^LC1^* mutant than control mice despite lower levels of cleaved ENaC (Figure 5a). The increased amiloride‐sensitive Na^+^ excretion is likely a consequence of enhanced Na^+^ delivery to the ASDN that results from decreased NCC activity in the *Sgk1^Pax8^* ^/^ *^LC1^* mice. Additionally, K^+^ secretion was decreased in the mutant vs. control mice after amiloride treatment (Figure 5b), which might indicate that ENaC‐mediated Na^+^/K^+^ exchange plays a role in the early kaliuresis, at least in the phase that requires ENaC activation which observed in this study 6 h after the start of the meal. The fact that ENaC‐mediated Na^+^ reabsorption is increased in the mutant mice may interfere with the interpretation of the HCTZ‐sensitive component of Na^+^ excretion as some of the Na^+^ that escapes the DCT due to NCC inhibition will be reabsorbed downstream, at least in part in exchange for K^+^. For that, we analyzed (Na^+^+K^+^) excretion as an additional indicator of NCC activity and found no difference between control and mutant mice (Figure 5d). No difference in urinary flow (Figure 5c) or water intake (Figure 5e) was observed between the genotypes. Thus, in the absence of SGK1, exaggerated Na^+^ delivery from DCT might compensate for the defect in ENaC cleavage and K^+^ secretion, enabling *Sgk1^Pax8^* ^/^ *^LC1^* mice to maintain K^+^ secretion upon acute high K^+^ challenge.

## 4 DISCUSSION

In this study, we found that kaliuresis is rapidly activated after ingestion of a K^+^ load by two sequential and overlapping processes. In the immediate phase, a rise in extracellular K^+^ immediately stimulates K^+^ excretion through a SGK1 independent process (see below). Our findings are different from studies involving mice with germ‐line deletion of SGK1 (SGK1^−/−^) that described reduced ability of the mutant animals to secrete intravenous K^+^ load over 30 to 60 min (Huang et al., 2004). It is conceivable that, the more profound phenotype in the global knockout involves some undefined extrarenal role of SGK1 in K^+^ homeostasis, or it is more likely that the long‐life absence of SGK1 has a profound detrimental effect on the potassium‐secretory machinery. Studying adult mice quickly after inducing the knockout prevents this.

In addition, our experimental design involves administration of K^+^ loading during the active period of the day to ensure better control of endogenous circadian rhythms, which may account for some of the discrepancy with previous studies in which the time of day, and thus the endogenous rhythmicity of K^+^ secretion, may have not been controlled.

Here, we found that, within 3 h of the K^+^ load, SGK1 becomes activated, as evidenced by the SGK1‐dependent phosphorylation of NEDD4‐2. The response is in agreement with our previous data showing aldosterone treatment in ADX rats, rapidly inducing SGK1 expression and NEDD4‐2 phosphorylation (Flores et al., 2005). Within 6 h after HKD, the increase in NEDD4‐2 phosphorylation coincides with an increase in proteolytic processing of ENaC, both of which are compromised in the *Sgk1^Pax8^* ^/^ *^LC1^* animals. We have previously demonstrated that ENaC ubiquitylation regulates its cleavage (Ruffieux‐Daidie et al., 2008), suggesting that the defect in ENaC processing in the *Sgk1* KO mice is likely a direct consequence of NEDD4‐2 activation. Consistent with our observation, Sorensen et al. (2013) found an increase α and γENaC cleavage 6h after K^+^ loading via gavage. However, we cannot rule out a K^+^‐dependent, aldosterone‐independent activation of ENaC and/or apical K^+^ channel under these circumstances. Indeed, recent studies have revealed that high extracellular K^+^ activates ENaC in aldosterone‐independent manner (Sorensen et al., 2019; Yang et al., 2020). Together, these data indicate that ingestion of a high K^+^ load rapidly induces a kaliuretic response in an early SGK1/NEDD4‐2‐independent phase, initiated by increased Na^+^ delivery from DCT to downstream segments, accompanied by an enhanced Na^+^/K^+^ exchange, and a later phase that requires SGK1 inhibition of Nedd4‐2, accompanied by increased ENaC cleavage.

Here, we observed that hyperkalemia is more severe after 6 h than 3 h of high‐K^+^ intake despite (a) the fact that the majority of food intake occurs during the first 3 h of food availability, and (b) the presumed elevation of aldosterone to facilitate K^+^ excretion. This surprising finding might be explained more likely by the delay between food consumption and the time required for the ingested and absorbed K^+^ to enter the circulation. Moreover, it is not unexpected that the ability of intracellular compartment to uptake K^+^ is maximal during the early phase of high‐K^+^ intake. This capacity might be reduced in later phases either because of reaching plateau or because the aldosterone‐mediated renal adaptations to secrete K^+^ load are now at play. The later assumption requires a cross‐talk between kidney and skeletal muscle during early kaliuresis, a such mechanism has not been yet investigated.

The activation of SGK1 after only 3h of the start of HK intake might involve aldosterone‐dependent and/or aldosterone‐independent mechanisms. Interestingly, the activation of SGK1 by mTOR is shown to be directly driven by an increase in extracellular K^+^ (Sorensen et al., 2019). It is conceivable that the first step of SGK1 activation during early kaliuresis involves K^+^‐induced phosphorylation cascade while a long‐term regulation of the kinase requires the aldosterone‐dependent increase in its protein level.

As early as 15 min after ingestion of the potassium‐rich diet, the aldosterone/SGK1 independent kaliuretic mechanism becomes active. Mediated by K^+^‐induced proximal natriuresis (Weinstein, 2017) and K^+^‐dependent NCC dephosphorylation in the DCT (Sorensen et al., 2013; Terker et al., 2015), the immediate kaliuretic process is stimulated as Na^+^ delivery to distal segments activates electrogenic sodium‐potassium exchange through the preexisting K^+^ secreting machinery (Buren et al., 1992; Chen et al., 1999; Loffing et al., 2001; Rabinowitz et al., 1985; Sorensen et al., 2013). A potassium‐sensing signaling pathway, consisting of basolateral membrane potassium‐sensing potassium channels, Kir4.1/5.1, and WNK‐SPAK kinases allows plasma K^+^ to directly, and immediately phospho‐activate NCC when K^+^ is low and inhibit NCC when K^+^ is high (Terker et al., 2015). Our data indicate that SGK1 in DCT interacts with the kinase‐cascade in the late phase to brake dephosphorylation. One may wonder about the significance of maintaining some residual phosphorylation of NCC during HK intake. We speculate that it safeguards against excessive K^+^ loss in the late phase when SGK1 has activated ENaC, and provides a “Brake‐Pedal” that anticipates the end of the meal. This can explain our surprising observation that potassium excretion reaches a plateau by 3 h even though ENaC is activated in late aldosterone‐dependent phase (3–6 h).

SGK1 is known to regulate NCC activity by suppressing NEDD4‐2‐mediated ubiquitylation of NCC (Arroyo et al., 2011; Ronzaud et al., 2013; Rozansky et al., 2009), and interfering with WNK4 and WNK1 signaling (Roy et al., 2015; Rozansky et al., 2009), but it is not clear how elevated K^+^ levels induce SGK1 in the DCT. Since SGK1 controls K^+^ balance, which in turn regulates NCC abundance and phosphorylation, it can be challenging to isolate a direct role of SGK1 in the DCT. We did not detect a difference in plasma K^+^ between SGK1 KO and control mice after an acute K^+^ load, making it likely that SGK1 is directly affecting NCC in the DCT. Transcription of the *sgk1* gene is activated by either the glucocorticoid receptor (GR) or the mineralocorticoid receptor (MR), depending on the cell type, relative expression of MR/GR, and the associated level of 11bHSD (Bostanjoglo et al., 1998; Buse et al., 1999; Chen et al., 1999; Funder J, 2017; Hunter & Bailey, 2015; Loffing et al., 2001; Nesterov et al., 2021). In the DCT2, 11bHSD is co‐expressed along with MR (Bostanjoglo et al., 1998), making plausible an indirect regulation of NCC phosphorylation involving high K^+^/aldosterone‐induced SGK1. In our previous studies (Al‐Qusairi et al., 2016; Faresse et al., 2012), we found higher aldosterone levels in the *Sgk1^Pax8^* ^/^ *^LCl^* vs. control mice under basal condition, and under aldosterone‐stimulating conditions including 2 days of high‐K^+^ diet or 4 days of low‐Na^+^ diet. Although aldosterone levels were not measured in this study, one may speculate that mutant mice used here exhibit higher aldosterone levels compared to WT mice, under basal and aldosterone‐stimulating conditions. Alternatively, an aldosterone‐independent MR signaling pathway, similar to one recently described in the ASDN, which might be mediated by glucocorticoid binding (Nesterov et al., 2021), may be involved.

Our diuretic challenge indicated decreased NCC activity which is consistent with a reduced NCC phosphorylation while the increase in ENaC activity despite an altered processing was unexpected. One may speculate that the observed increased in amiloride‐sensitivity is caused by increased Na+ delivery to ENaC expressing segments due to more severe dephosphorylation of NCC in the mutant mice. Moreover, AngII is a potent stimulator of ENaC function (Peti‐Peterdi et al., 2002; Zaika et al., 2013) and, a compensatory increase in AngII levels has been described upon decreased aldosterone signaling (Todkar et al., 2015). Interestingly, Yang et al. have recently demonstrated a dissociation of ENaC activity from channel processing in the total SGK1^−/−^ model, where ENaC function was maintained under HKD and increased under control diet despite defected cleavage in the mutant compared to control mice, which is in agreement with our observations (Yang et al., 2017). Alternatively, a residual SGK1 expression in the *Sgk1^Pax8^* ^/^ *^LCl^* might account, at least in part, for the mild physiological effect observed in this study. Indeed, in our previous report (Al‐Qusairi et al., 2016; Faresse et al., 2012), we detected up to 60% and 50% deletion of the sgk1 mRNA respectively, while SGK1 protein was decreased by 95% and 90%, respectively. Since SGK1 is ubiquitously expressed, the remaining mRNA and protein are more likely to be attributed to non‐tubular cells. However, despite the uncomplete deletion of SGK1, mutant mice exposed to high‐K^+^ diet for 2 days develop hyperkalemia due to a defect in renal K^+^ secretion (Al‐Qusairi et al., 2016) suggesting that the residual SGK1 protein is unable to support chronic K^+^ excretion. However, we cannot rule out the possibility that acute K^+^ clearance requires lower expression level of the kinase than the chronic K^+^ clearance.

In conclusion, the current study revealed (a) the early phase of acute K^+^ secretion is independent of SGK1 action. (b) A SGK1‐dependent phase starts after 3h of K^+^ intake in which SGK1 function is required to ensure an adequate activation of ENaC while driving residual phosphorylation of NCC to more likely limit excessive K^+^ loss to anticipate the end of the meal.

## 5 LIMITATIONS

It is worth noting that our study focused on young mice (7–8 wk‐old). At this growing phase, the addition of new cells results in an increase of the K^+^‐rich intracellular compartment. One may expect that, cellular K^+^ uptake might be more active in young mice, which might affect the homodynamic of K^+^ redistribution between the intracellular and extracellular compartments.

In addition, ENaC activation was evaluated at several time‐points in this study by measuring the cleaved form of αENaC, which might not be the most accurate measurement in this context. Indeed, the regulation of ENaC is complex since it requires phosphorylation (Shimkets et al., 1998), apical localization (Firsov et al., 1996; Frindt et al., 2008), and cleavage (Awayda et al., 1997; Bruns et al., 2007). Indeed, the most accurate analysis in the context of our study is a direct measurement of the channel activity by patch‐clamp.

## CONFLICT OF INTEREST

No conflicts of interest, financial or otherwise, are declared by the authors.

## Supporting information



Supplementary Material
