# Supplementary figures and images for: Does the early aldosterone‐induced SGK1 play a role in early Kaliuresis?
Source: Physiol Rep. 2022 Feb 28;10(4):e15188. doi: 10.14814/phy2.15188 (PMC8883148; doi:10.14814/phy2.15188)

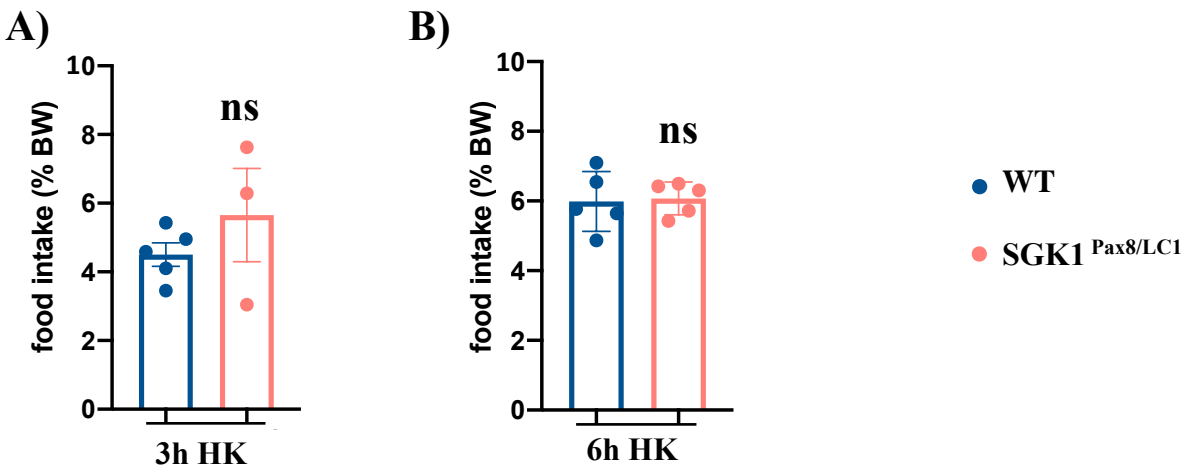

Supplement: Supplementary file 1 — Supplementary Material [file PHY2-10-e15188-s001.pdf]
